# Farmers’ Heterogeneous Willingness to Pay for Farmland Non-Market Goods and Services on the Basis of a Mixed Logit Model—A Case Study of Wuhan, China

**DOI:** 10.3390/ijerph16203876

**Published:** 2019-10-12

**Authors:** Xin Yang, Anlu Zhang, Fan Zhang

**Affiliations:** 1 College of Land Management, Huazhong Agricultural University, Wuhan 430070, China; 2 School of Agriculture and Environment, The University of Western Australia, Crawley 6009, Australia

**Keywords:** farmland non-market value, willingness to pay, mixed logit, preference heterogeneity, farmers

## Abstract

The exploration of different stakeholders’ heterogeneous willingness to pay for farmland ecological value is a fundamental part of understanding the total value of farmland protection and designing a scientific farmland protection policy. Unlike the homogenous assumption used in the previous studies, the mixed logit model of choice experiment method was applied to estimate respondents’ heterogeneous willingness to pay for farmland non-market value (represented by farmland area, farmland fertility, water quality, air quality, species richness, and recreational value) in this study. Data came from a sample of 289 farmers in Wuhan, China who were face-to-face interviewed. Results showed that: (1) Farmers were unsatisfied with the status quo of the present farmland ecological environment and were willing to pay to preserve all the attributes of farmland non-market value. (2) Farmers had a heterogeneous preference for the status quo and recreational value—the error variances of these two attributes were both significant at the 1% level, and their willingness to pay for the farmland non-market value in Wuhan was 1141.88 Yuan/hm^2^. (3) Farmers’ cognition degree of farmland importance and whether respondents bought medical insurance or not had significant impacts on their willingness to pay. The results can provide the basic foundation for accurate valuation of farmland non-market services, help farmland regulators make the right farmland conversion decisions, and improve the resource allocation efficiency of local financial expenditure during farmland protection in Wuhan.

## 1. Introduction

Farmland provides humans not only with economic value, such as food and fiber material, but also with a secure range of environmental public services, such as open space conservation, fresh air, flood protection, preserving wildlife habitat, and biodiversity [1,2,3,4]. However, because of its insignificant value relative to other land-use types, a large amount of farmland is occupied every year by construction land during the rapid urbanization process in China [2,5,6,7,8]. Moreover, large amounts of chemical fertilizer and pesticides have been abused by farmers in China to guarantee the national grain production and effective supply of agricultural products. This kind of high input–output tillage model has led to serious environmental problems, such as soil erosion, food security crisis, and unsustainable farmland development, among others [9,10,11,12], imposing severe threats to the safety of agricultural products, drinking water, and the sustainable development of society. Thus, the means to which environmental non-market goods and services generated by farmland are quantified in monetary terms is the most fundamental part of protecting farmland and solving the above mentioned problems in China.

Recognizing and estimating respondents’ heterogeneous preference for the non-market goods and services of farmland is crucial for the success of a farmland ecological compensation program [13,14]. Farmers play multifunctional roles in the farmland protection process. In addition to being providers of various farmland ecological services, farmers are also consumers of the above farmland non-market goods and services. They need to share the cost of improving farmland ecosystem. Therefore, their participation is one essential part for the valuation of farmland non market goods and services [2]. It is critical to understand the crucial role of farmers by exploring their heterogeneous willingness to pay for farmland non-market value.

As we have explored the citizens’ preferences for the farmland non-market value in our previous studies [8,15]. The aim of this study was to estimate respondents’ heterogeneous valuation of farmland ecological services and the associated social-economic characteristics that have impacts on this valuation work, coming from the perspective of farmers in Wuhan, China. The examined farmland non-market value contains farmland area, farmland fertility, water quality, air quality, species richness, and recreational value.

As the above components are not traded in markets and play a fundamental role in guaranteeing food security and ecological environment in China, a hypothetical market is need for the valuation work [16]. Stated preference technique that can account for farmland non-market value was used in this paper to analyze the choice modeling data. Quantitative monetary trade-off information between cost and farmland non-market benefits can be obtained from the outcomes, which was of fundamental help to the decision makers and the public in understanding the whole value of farmland, making the right decision on whether farmland should be converted into urban land or not, and designing farmland non-market value protection policies that allied more with farmers’ heterogeneous preference [2].

The study is arranged in six parts: the literature review is done in Section 2, and Section 3 begins by describing the methodology and its evolutionary path, introducing the study area and the data collection. Section 4 reports the results of the study by applying the mixed logit model of choice modeling. The interpretation and policy implications of the results are discussed in Section 5. Finally, the last section (Section 6) states the limitations and conclusions of the research.

## 2. Literature Review

Prior to extinction, many continent valuation studies demonstrate significant public interest in protecting the environmental non-market amenities provided by farmland [17,18,19,20]. Although the above research has provided a sound basis for non-market valuation estimation, it has been criticized for its emphasis on the value of the whole farmland, and different attributes of farmland are seldom considered, which may lead to a bias in the estimation results. This can be avoid by choice modeling, which not only can solve the bias associated with other stated preferences techniques [13], but also can allow the researcher to estimate respondents’ tradeoffs between attributes simultaneously [21]. Originating in the marketing and economics of transport, choice modeling was soon widely applied to evaluate the non-market values of different kinds of environmental goods and services in the last 20 years, such as river value [22], gene modified food [23], rural tourism [24], clean energy [25], solid waste management [26], marine fishing [27], forest [28,29], mine site rehabilitation [30], water [3,31], and agricultural land [9,14,19]. Moreover, individual’s homogeneous preference has been criticized for its bias leading to estimation. Therefore, accounting for heterogeneity in choice modeling can help researchers express the respondents’ real preference better and gain an unbiased estimating result. There were issues associated with the different preferences between social groups for non-market goods and services in previous valuation literature [32,33,34,35,36].

Management of farmland resources always involves difficult decisions [10]. Present farmland protection began to take all its attributes into consideration rather than solely focus on the whole state of farmland [15,37,38,39,40]. There has been an attempt to integrate farmland protection and local residents’ participation. Thus, the means to which residents’ preferences can be revealed along with their heterogeneous willingness to pay for the ecological goods and services generated by farmland is the key part of farmland non-market evaluation. Despite being the main providers of farmland environmental services, farmers are also consumers of farmland non-market goods and services [41]. It should come as no surprise that farmers should share the cost of preserving the farmland ecological services—their preferences and participation are important for the conservation of farmland non-market value [9,10]. Along with citizens, farmers’ preferences for farmland ecological value are likely to vary across individuals, which should be taken into consideration during the farmland management process [42].

The above findings reveal that different respondents’ demands for farmland non-market value exist, also showing that the differences between social groups are essential. Therefore, by including of attributes of ecological goods and services in choice modeling, the process of taking respondents’ heterogeneous preference into consideration is highly recommended for a more accurate estimate [43]. However, as far as we are concerned, research that has explored farmers’ heterogeneous willingness to pay for farmland non-market values was either applied by contingent value method (CVM) or choice modeling (CE) with homogenous preference assumption [7,8,44]. Literature that assumes respondents’ preferences vary among every individual, mainly concentrating on the perspective of citizens [15], and very few previous studies have applied mixed logit model to account for this kind of heterogeneity coming from the perspective of present-day China.

This paper can make contributions in three ways: First, it estimates the farmland non-market value from the perspective of farmers, who play a multi-role in farmland protection. Second, it adds to the growing literature that has applied mixed logit model to explore heterogeneous preference for farmland non-market value in China. Third, it has an environmental policy design through estimating farmer valuation of various components of farmland.

## 3. Methods, Study Area, and Data Collection

### 3.1. Methodology

Developed originally from transportation research [45], choice modeling is an emerging approach. It rooted in random utility theory used evaluate the non-market value of environmental commodities with more than one attribute [22,40,46,47,48,49,50]. It assumes that consumers always rationally choose the option that can provide them with the highest utility. However, respondents’ behavior varies across individuals. To explore the heterogeneity of their preference, mixed logit model is applied in this paper to capture more realistic non-market valuation estimation. Mixed logit (ML), which is also called random parameters, assumes that marginal utilities of individuals are not constant but vary across a sample. The typical approach to an ML is centered on random coefficients. It relaxes the restrictive “independence from irrelevant alternatives” assumption and allows every individual to have their own preferences, that is, the parameters from each decision maker are independent of each other. The random parameters for the particular individual should be constant across the repeated choices, which can be captured for the heterogeneity identification. The parameters are usually assumed to follow a normal or lognormal (limited to non-negative range) distribution. After selecting the distribution, parameters can be estimated through simulated maximum likelihood, that is, Hole’s Stata module mixlogit [33].

The ML model is centered on random coefficients. It assumes that there is a distribution of marginal utilities across the sample. The utility that individual *n* derives from choosing alternative *j* (*U_nj_*) is given by Equation (1):

(1) $$U_{nj}=\beta_{n}X_{xj}+\varepsilon_{nj}$$

where *β_n_* is a vector of coefficients of observed variables for individual *n*, *X_nj_* is observed variables relating to individual *n* and alternative *j*, and *ε_nj_* is the unobserved utility for individual *n* and alternative *j*.

In the ML model, the coefficients vary over individuals within a population of density f(β/θ). f(β/θ) is a function of the mean and covariance of the population betas, represented by parameter *θ*. The primary difference between homogenous and heterogeneous preference is that *β* is allowed to vary over individuals.

Assuming the probability is conditional on *β_n_*, respondent *n* choosing alternative *i* can be expressed by Equation (2):

(2) $$L_{ni}(\beta_{n})=\frac{e^{\beta_{n}{}^{\prime }x_{ni}}}{\sum_{j}e^{\beta_{n}{}^{\prime }x_{ni}}}$$

This is the conditional logit equation [50]. As *β_n_* is unconditional on *β*, and the utility is assumed to be linear in parameters, the integral of *L_ni_* (*β_n_*) over all possible *β’_n_* therefore becomes the unconditional choice probability. The unconditional probability integrated over the distribution of *β* can be written as Equation (3):

(3) $$P_{n}(\theta )=\int S_{n}(\beta )f(\beta /\theta )d\beta$$

This probability is a weighted average of the stand logit formula, and *β* is evaluated at different values determined by the density *f* (*β/θ*).

Not only individual-specific and alternative-specific explanatory variables are allowed to fitting models when specification is general—as the distribution function is specified by the research, typically as a normal or lognormal distribution (Train 2009), its log likelihood value can be obtained from the $LL(\theta )=\sum_{n=1}^{N}L_{n}P_{n}(\theta )$. However, it can only be solved by applying simulation methods rather than analytical method, and log likelihood of the simulated form can be written as Equation (4):

(4) $$SLL(\theta )=\sum_{n=1}^{N}In\left(\frac{1}{R}R\sum_{r=1}S_{n}(\beta^{r})\right)$$

where *R* is the replication times and *β*’ is derived from *f* (*β/θ*).

Specifically, one should ideally attempt to model all attributes as random parameters, but in reality this often is not feasible. Even for a small number of attributes, there are distribution considerations, as discussed above, as well as consideration of possible correlations between choice situation (i.e., for *t* repeated choices by an individual) attributes, or an attribute’s parameters [21]. Consideration of all of these factors makes estimation lengthy. Estimation of willingness to pay (WTP) also becomes complicated in an ML. Partworths are no longer a simple ratio of two parameters but a function of the distribution of the random parameter distribution, it can be calculated by applying the mean and standard deviation estimates [21].

(5) $$WTP_{i}=\frac{\beta_{i}}{\beta_{7}}$$

where *WTP_i_* is farmers’ willingness to pay for attribute *i*, *β_i_* is their coefficients, and *β_7_* is the coefficient of cost. The consumer surplus can be written as Equation (6):

(6) $$CS=\frac{1}{\beta_{r}}|In\sum \exp V^{0}\;-\;In\sum \exp V^{1}|.$$

### 3.2. Study Area

**Figure 1:**
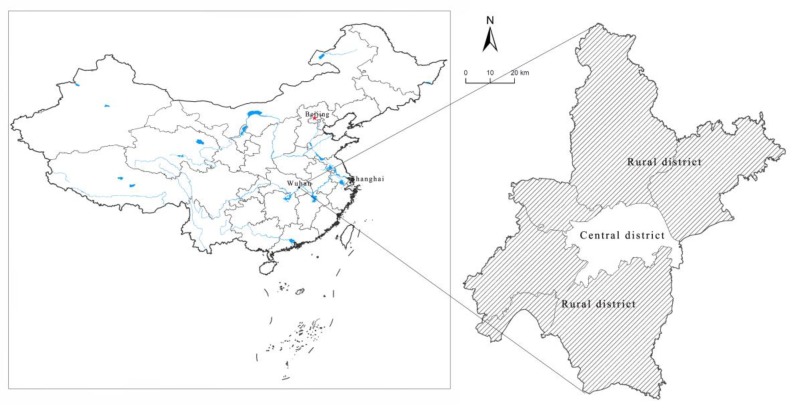
Regional plan of the study area.

The study was carried out in one of the largest farmland area of Hubei province, Central China (Figure 1), which is divided into seven central districts and six rural districts (Figure 1). It is located at the intersection of the Yangtze River and Han River, and plays an important role in China’s largest grain producing area—Jianghan Plain in China. It covers an area of 8494 km^2^, and 35% of this is farmland. There is a diverse array of ecological systems in Wuhan, of which farmland is the most important one because of its absolute advantage in quantity terms. Therefore, Wuhan is a type of farmland ecosystem, which is especially true for the seven rural districts. However, because of the rapid urbanization process and farmland protection pressure from the central government, serious farmland loss, and overuse of chemical fertilizer and pesticides, the farmland ecological system has suffered serious deterioration. This area not only proves serious conflicts between farmland protection and economic development, but also is a quintessential representative of large cities worldwide that have heavy urbanization pressure.

Attributes that needed to be protect include the area and fertility of farmland, plant and animal resources as well as their habitats, water conservation in the main rivers and lakes in Wuhan, air quality, recreational value brought by the farmland landscapes, and open spaces. Moreover, Wuhan is also the central city of the Wuhan Agglomeration Area, which is one the two national pilots developed to construct an “environmental friendly and resource saving” society. All of the above makes it a highly typical yet unique area for farmland non-market valuation research. Because of public participation’s fundamental role in the design of a successful farmland protecting policy, the estimation of farmers’ heterogeneous willingness to pay for the afore mentioned farmland non-market goods and services and the exploration of its potential influencing factors in their preferences for these non-market values can provide a basic scientific foundation for policy-makers from the farmland protection department.

### 3.3. Data Collection

In choice modeling, identifying appropriate attributes is of great importance. Farmland not only provides people with food and material, which are of great importance to national food security, but also acts as an ecological barrier in protecting rural landscape and species richness [8]. However, the rapid urbanization process and the over-dosing of chemical fertilizers and pesticides used in farmland production make the farmland ecosystem become vulnerable. With reference to previous research [1,15,44], we selected farmland area, farmland fertility, water quality, air quality, species richness, and recreational value as the 7 attributes to describe the farmland non market values. The design of attribute level affects the accuracy and validity of the estimation directly. Therefore, researchers must ensure that selection of levels should be determined via attributes’ current level and its optimal state that can be obtained after the implementation of the eco-compensation. These levels should also be consistent with the practical situation and easy for the respondents to understand [14,51]. Moreover, continuous variables are likely to get a more precise estimation result. Because of the lack of data, the levels of farmland fertility and air quality were both set as discrete variables, but for the farmland area, water quality, species richness, and recreational value were set as continuous variables in this study.

In particular, the levels of cost showed in this paper were selected on the basis of a previous CM study and the pilot survey [8]. Through the pilot survey, we found that the “50 Chinese Yuan per person per year” has the highest probability of being selected, and the highest amount individuals are willing to pay is “151–200 Yuan per person per year”. Therefore, a combination of “0, 50, 100, and 200” was selected as the level costs to be paid. Table 1 shows all the level of attributes and their coding names.

According to the CM design, those attributes and their levels can generate 4^6^ × 5 = 20,480 combinations. In a multi-attribute multilevel choice set, the identification and efficiency of the estimates crucially depends on the choice of experimental design [52]. Orthogonal test was employed to reduce the final number of combinations. Furthermore, with respect to cognitive burden and complexity issues, the literature suggests three or four alternatives as being optimal [53,54]. In the end, we had 20 choice sets in total. In order to reduce the complexity and length of the questionnaire, 20 choice sets were blocked into 4 versions, each with the respondent only need to answer 5 choice sets. In each choice set, respondents were asked to choose between three alternatives: status quo, improving option A, and improving option B. Table 2 shows a typical scenario presented to farmers.

The questionnaire consisted of three parts. In the first part, after giving the background information, nine functions of farmland were presented to respondents, where in they were asked to indicate their degree of agreement. The second part contained seven choice sets. The concept of choice questions were introduced, and respondents were asked to make a choice from the following three options in each choice set: SQ (the status quo), option A (farmland non-market improvement option), and option B (a different farmland non-market improvement option).The third part of the questionnaire contained the questions about the respondent’s socio-demographics, such as age, gender, education level, and income, among other factors.

Generally, the data’s accuracy was dependent on the budget available for the survey. However, Schaeffer provides a formula to calculate the minimum sample size. According to the formula, statistic principle (5% error), and interest group, we needed at least 270 samples [55]. 10 trained enumerators majoring in land resource management were employed to do the face-to-face survey in January 2015. A total of 320 questionnaires were completed in rural areas of the city (see Figure 1), with 80 of each version. In total, 289 farmers responded, and the overall responding rate was90.31%, and different rates for version 1(87.50%), version 2 (88.75%), version 3 (90.00%), and version4 (95.00%) were found.

## 4. Results

Stata 12.0 was used to fit the mixed logit model, and socio-demographics were included in the utility function either through interactions with the ASC (alternative specific constant) or as interaction terms with the choice attributes.

### 4.1. Definition of Variables

Warming up effect is highly valued in CM studies [21]. Farmers’ cognition of farmland multifunction was investigated, as it can help respondents have a basic understanding of the farmland protection problems in this paper, which is useful for the choices they make. Farmers’ socio-demographics were also obtained, namely, their age, gender, educational level, and income, among other factors. These can help achieve a more precise estimation, as well as indicating how well the sample represents the population. In particular, variables that were not significant in the models were not included in the final model specifications; thus, only those that were significant are listed in Table 3.

For farmers’ cognition of the importance of the five non-monetary attributes in Table 3, it was obvious that they held the opinion that water quality was the most important factor, followed by air quality, species richness, farmland fertility, and farmland area, where as the emphasis on recreational value was relatively weak.

As for the farmers’ basic socio-demographics, the average education level of farmers was relatively low (6.221 years), the family size was about four members, 14.64% of them had debt, and 93.48% of the farmers had medical insurance. A total of 55.62% of them were willing to do farmland work in the following 5 years. The annual net income of the farmers’ household was 35.204 × 10^3^ yuan. Only 19.28% of the farmers were familiar with the prime farmland protection policy (only those who were significant were listed in Table 3).

### 4.2. Result of Mixed Logit Model

As discussed in Section 4.1, there were various estimation techniques that could be prescribed as extensions to the basic homogenous models. Mixed logit (ML) model is one of the techniques that features well with reality, as it can account for heterogeneity across individuals by including an individual specific error term. Results of the ML model are listed in Table 4.

The ML model results showed that overall there was a pro-conservation trend toward the farmland non-market attributes, as shown by the positive coefficients. In particular, the information supported the existence of heterogeneity in the data. The ML model indicated that respondents had significantly heterogeneous preferences for the attributes of status quo and recreational value (Recv). More importantly, despite the mean value for status quo being negative, the value of stand error indicated that a small proportion of the population chooses the status quo. Moreover, it was significant at the 5% level. The majority of the samples were estimated to show no significant preferences for the recreational value (Recv), although some of the farmers showed positive preferences for this attribute, and its stand error was significant at the 1% level.

In particular, Table 4 also indicates that respondents were inclined to choose the conservation alternatives rather than the status quo, as seen by the negative ASC coefficient for the ML model. Moreover, there were several significant interactions on the ASC parameter, and there was a baseline preference towards choosing the conservation programs in favor of the status quo for any given level of attributes. The preference was reinforced by farmers who believed that they will still do farm work and those that had debt from banks. Respondents’ preference for the recreational value (Recv) was influenced by those who did not have medical insurance.

For the attribute of farm area, its coefficient was positive (0.6174) and was significant at the 1% level, which implied that farmers wanted to slow down the farmland loss situation and wanted to have larger area of farmland. Farmers who realized the importance of farmland fertility (Farmf) showed a significant positive preference for higher levels of this attribute that was significant at the 5% level. Moreover, farmers’ preference for air quality (Airq) and species richness (Species) were positive (0.3446 and 0.06, respectively) and statistically significant (1% level and 5% level, respectively). The values derived were dependent upon Equation (7) and Table 4, and the results are listed in Table 5.

### 4.3. Estimates of Part-Worths

According to the ML model, the estimated coefficients were used to estimate the rate at which respondents were willing to tradeoff one attribute for another. Assuming the other attribute as equal, we can evaluate the marginal value of an attribute. The marginal value can be explained as: in order to improve the attributes by one level, how much the farmers were willing to pay.

Table 5 shows that the marginal values for all of the attributes are positive, implying that the respondents have a positive WTP for an improvement in the three attributes. According to the definition of the attributes and their levels, the interpretation for discrete attributes and continuous attributes are different [56]. Farmland fertility and air quality are discrete variables, improving those two attributes by one level were 28 Yuan and 293 Yuan, respectively. With regard to the attributes of the farm area, water quality, species, and recreation value, which are continuous variables, for the marginal value measure for the value of a 1% improvement in the attribute for the continuous variables, respondents were, respectively, willing to pay 105, 19,53, and 107 Yuan annually.

The definition of scenarios was the fundamental part for farmland non-market values based on farmers’ WTP. In this paper, the annual WTP of a typical agricultural household for the farmland non-market values was defined as the value difference between the status quo and the best option for all choice sets. It was approximately 604.61 Yuan per person annually. In some similar studies, respondents’ willingness to pay was about 164.30–624.44 Yuan per person per year [8,15,44]. The willingness to pay of respondents in this survey was moderate and reasonable. Therefore, the total WTP from farmers can be performed by multiplying farmers’ average WTP by the number of households in Wuhan in2015 (77.20 × 10^4^ Yuan). By dividing the whole farmland area, the annual total WTP for the farmland non-market value per hectare was1141.88 Yuan.

## 5. Discussion and Policy Implication

### 5.1. Discussion

This research has the following three merits: (1) It contributes to the existing CM application literature. Unlike the previous homogenous preference assumptions, it is also one of the few studies of farmland non-market valuation protection that uses the ML model to analyze the heterogeneous choices of respondents [35,49,57]. This study provides researchers with a further way to explain the potential heterogeneity from the farmers’ perspective. (2) It can provide policy-making aid for farmland protection. During the rapid urbanization process, policymakers face trade-offs between economic development and farmland protection. Accurate market valuation estimation can help the policymakers understand the whole value of farmland correctly and make the right decisions when facing farmland conversion issues. Moreover, the CM model gives the different values of every farmland non-market value attribute; thus, policymakers can allocate the limited capital to the attribute that has higher weight in their utility function (e.g., air quality), thus improving the resource allocation efficiency of local financial expenditure. (3) It can fulfill the research field of farmland ecological compensation. Despite farmers suffering economic loss during the farmland protection process, they also need higher farmland non-market value, which includes more farmland area, higher farmland fertility, and better water quality, among other factors. Moreover, the latter factor is also neglected in farmland ecological compensation criteria research.

Despite the contributions of this study, limitations also exist. They are listed as follows: (1) The data of the study was collected in 2015, which is not recent. The data is from the last funding, which contained a series of research in 2007, 2012, 2013, and 2016 [7,8,15,44]. Farmers’ attitudes toward farmland protection only changed a little. The contribution of this study was to explore the famers’ heterogeneity during farmland non-market value by applying choice modeling; thus, the timing of the data’s impact on the conclusion is limited. Moreover, the cost of data recollection is very high, which makes recollection work more difficult. (2) The second limitation lies in the small sample size of this study. Though the minimum sample size was satisfied in this study, a large sample is still needed for the stated preference technique. As larger sample is the precondition required to achieve results that have universality and applicability. (3) Despite the recent and rapid development of the CM method, its technique is still not mature. Aside from the need to take care in framing the context of study topic and attributes, one limitation of this study was the hypothetical bias and embed bias [58], the same as other stated preference studies. (4) More levels of attributes would help to gain a more accurate estimation, but recognition burden must be taken into consideration, and thus only four levels were set for non-market attributes and five levels for monetary attributes. (5) Lastly, we only checked the prime farmland protection policy’s effect on farmers’ willingness to pay for farmland services, which was proven to be insignificant in the end. However, other existing or non-existing environmental policies have been implemented in some areas of China, such as conservation tillage promotion in Northeast Plain [59], farmland ecological compensation mechanism in some developed cities [60], and carbon trading pilot [61]. Despite the above environmental policies being employed either at the governmental or enterprise level, they may still affect farmers’ willingness to pay, which we neglected to add into our questionnaire.

### 5.2. Policy Implication

First, in light of the severe threat of farmland goods and services having drawn the attention of both the government and public, strict farmland protection policies (i.e., *Land Use Planning*, *Primary Farmland Protection Areas,* and *Main Function Planning*) have been criticized by many scholars and development practitioners as ineffective because of their emphasis on economic output and their neglect of non-market value [15]. Thus, understanding farmers’ heterogeneous preference is of great importance to quantify farmland non-market value correctly. Accurate farmland non-market valuation can help regulators make the right decisions when facing farmland conversion issues during rapid urbanization process.

Moreover, farmland ecological programs designed on the basis of farmland’s whole value has been widely applied in western countries to provide compensation for farmland’s role in guaranteeing rural open space and various farmland ecological services [9,62,63], and it is still increasing in many developing countries who are trying to seek a balance between economic development and environmental protection [64]. The farmland ecological compensation mechanism has also been put forward by the central and local governments in China, and developed regions such as Suzhou, Chengdu, Foshan, and Shanghai have begun to explore the value of their own farmland ecological compensation mechanism. Therefore, the evidence of this study can provide the basic foundation for valuation of farmland non-market services, as well as the construction of farmland ecological compensation mechanism in Wuhan, the largest city in Central China.

Other institutional innovations of environmental policies have been explored in some Chinese ecologically sensitive areas, such as conservation tillage promotion in Northeast Plain [59], river chief policy in Wuxi, Jiangsu Province [65], and carbon trading pilot [61]. Despite being one of the seven pilots, the carbon trading system is mainly implemented at the enterprise level, which has no close correlation with farmers. Jianghan Plain as been pointed out as one of the pilots for conservation tillage promotion, and a stricter environmental tillage policy has been put forward by the Ministry of Agriculture and Rural Affairs, which can be one of the causes for farmers’ willingness to pay for the farmland ecosystem. River chief policy can be introduced into the farmland protection field, but it still needs time for the farmers to accept it.

## 6. Conclusions

Aside from its social, cultural, and aesthetic values, farmland resource protection has non-market value [1]. This paper investigated farmer valuation of farmland non-market value in the biggest city in Jianghan Plain—Wuhan. A choice experiment survey was conducted with a random sample of 289 farmers from the rural districts of Wuhan. Compared with the traditional homogenous assumption, a mixed logit model was estimated in order to identify the respondents’ heterogeneous preference for the different attributes of farmland non-market value.

The results of this study implied that the farmers were aware of the importance of the farmland non-market value attributes. According to Table 3, the importance degree for water quality was 4.247, followed by air quality (4.121), species richness (4.090), farmland fertility (3.900), farmland area (3.340), and recreational value (3.043). Furthermore, Table 4 indicates that farmers dislike the status quo, and that 75.04% of them were willing to pay for alternative farmland improvement outcomes in Wuhan, China. Overall, the main purpose of this study was to assess farmers’ heterogeneous willingness to pay for farmland non-market value. According to the results of the econometric modeling, it was revealed that some interesting divergences on the heterogeneous preferences for status quo and recreational value preferences existed among the different groups. Moreover, preferences’ heterogeneity was also affected by some of their socio-economic factors, as those who will not do farm work in the following 5 years or do not have debts were more likely to choose the status quo. Farmers’ WTP for farmland fertility was also affected by their cognition degree on the importance of farmland non-market value. Medical insurance had a significantly positive impact on respondents’ WTP for recreational value.

As can be seen, the results of the mixed logit model of choice modeling in Table 5, the farmers’ estimated marginal value of the six attributes were all positive and significant. For the farmland fertility and air quality, which were discrete variables, their marginal value was, respectively, 28 Yuan and 293 Yuan annually. As for the attributes that belong to continuous variables, the willingness of farmers was 105 Yuan for farmland area, 19 Yuan for water quality, 53 Yuan for species richness, and 107 Yuan for recreational value. According to Equation (6), the consumer surplus (CS) of farmers can be defined as the difference between the choice set with every attribute at their highest level and the choice set with every attribute at their lowest level. Therefore, the total WTP from farmers was604.61 Yuan annually, and the farmland non-market value can be calculated at 1141.88 Yuan per hectare (paying rate 75.04%, 77.20 × 10^4^ household, 30.72 × 10^4^ hm^2^ farmland).

## Figures and Tables

**Table 1:** The levels of attributes and their codes.

Attributes	Level	Code
Amount of farmland	575, 576, 577, 578	Farma_4,3,2,1
The soil fertility and natural capability of farmland	4, 3, 2,1	Farmf_4,3,2,1
Proportion of qualified water from the main river’s section	70%, 75%, 80%, 85%	Waterq
An index used to show how polluted the air is	4, 3, 2, 1	Airq_4,3,2,1
The number of different species represented in the farmland ecological community	2500, 2505, 2510, 2515	Species
The value of farmland’s ability to provide people with enjoyment, amusement, or pleasure (1000 Yuan)	¥5, ¥5.5, ¥6, ¥6.5	Recv
How much is your family willing to pay to preserve the above values generated by farmland annually? (Yuan)	0, 50, 100, 150, 200	Cost

Note: levels with bold and underlined are the present level of each attribute.

**Table 2:** An example ofa choice set.

Attributes	Status Quo(S)	Option(A)	Option (B)
Farmland area	Farma_1	Farma_2	Farma_1
Farmland fertility	Farmf_3	Farmf_1	Farmf_2
Water quality	Waterq_4	Waterq_1	Waterq_1
Air quality	Airq_4	Airq_1	Airq_1
Species richness	2500	2500	2505
Recreational value	5000	6000	5500
Annual cost to your family	0	100	150
	I would like to choose A (), B (), or S for status quo ().		

**Table 3:** Definition of variables and their importance degree.

Variable	Definition	Means
Waterq *	The importance degree of water quality	4.247
Airq *	The importance degree of air quality	4.121
Species *	The importance degree of species richness	4.090
Farmf *	The importance degree of farmland fertility	3.900
Farma *	The importance degree of farmland area	3.340
Recv *	The importance degree of recreational value	3.043
Debt	Whether they have debt or not (yes = 1, no = 0)	0.146
Farm5	Whether they have done farmland work or not in 5 years (yes = 1, no = 0)	0.556
Medical	Whether have medical insurance or not (yes = 1, no = 0)	0.935

Note: variables with * are measured on a five-point Likert scale, where1= not at all important and 5=very important.

**Table 4:** Results of mixed logit model. ASC: alternative specific constant.

Variables	Coefficient	95%CIs	Coefficient	95%CIs
Mean			SD	
ASC	−13.2497 ***	−22.04, −4.46	23.61 ***	10.48,36.70
ASC*Farm5	−6.54 ***	−10.95, −2.14		
ASC*Debt	4.61 **	0.21, 9.01		
Farma	0.6174 ***	0.34, −0.89		
Farmf	0.18	−0.35,0.71		
Farmf*Importance	0.12 **	0.005, 0.232		
Waterq	0.02	−0.04, 0.08		
Airq	0.35 ***	0.10, 0.59		
Species	0.06 **	0.01, 0.12		
Recv	0.74	−1.01, 2.49	4.30 ***	1.96, 6.64
Recv*Medical	−1.72 *	−3.63, 0.19		
Cost	−0.018 ***	−0.024, −0.012		
**Summary Statistics**				
Log-likelihood	−1294.75			
Prob>chi^2^	0.0000			
LR chi^2^(2)	77.62			
Observations	3864			

Note: “*” “**” “***” indicates significant level at 10%, 5%, 1% level.

**Table 5:** Part-worths estimated at their mean value.

Attribute	Marginal Value	Z	*P* > |z|	95% CI
ASC	−232 **	2.7600	0.0220	−397,67
Farma	105 ***	4.9900	0.0100	64,146
Farmf	28 **	0.6000	0.0430	−63,119
Waterq	19 ***	0.7600	0.0020	−30,69
Airq	293 **	3.0100	0.0100	293,484
Species	53 **	2.6200	0.0440	13,92
Recv	107 **	1.5200	0.0420	−31,244

Note: “*” “**” “***” indicate significance level at 10%, 5%, 1% levels; part-worths listed in Table 5 are calculated at their mean value.
